# Metformin modifies plasma microbial-derived extracellular vesicles in polycystic ovary syndrome with insulin resistance

**DOI:** 10.1186/s13048-024-01444-x

**Published:** 2024-07-02

**Authors:** Liping Hu, Guolin Hong, Jingzhi Li, Mengkun Chen, Chih-Jung Chang, Po-Jen Cheng, Zhimei Zhang, Xinli Zhang, Huiping Chen, Yingting Zhuang, Yuqin Li

**Affiliations:** 1 Department of Laboratory Medicine, Xiamen Chang Gung Hospital Hua Qiao University, Xiamen, 361028 P. R. China; 2 The Third Clinical Medical College, Fujian Medical University, Fuzhou, P. R. China; 3 Department of Laboratory Medicine, Xiamen Key Laboratory of Genetic Testing, The First Affiliated Hospital of Xiamen University,School of Medicine, Xiamen University, Xiamen, 361005 P. R. China; 4 Department of Obstetrics, Xiangya Hospital Central South University, Changsha, P. R. China; 5 Department of Gynecology and Obstetrics, Xiamen Chang Gung Hospital Hua Qiao University, Xiamen, P. R. China; 6 School of Medicine, Hua Qiao University, Quanzhou, P. R. China; 7 School of Pharmacy, Fujian Medical University, Fuzhou, P. R. China; 8 Medical Research Center , Xiamen Chang Gung Hospital Hua Qiao University, Xiamen, P. R. China; 9 Drug Hypersensitivity Clinical and Research Center, Department of Dermatology, Chang Gung Memorial Hospital, Taoyuan, Linkou, Taiwan

**Keywords:** PCOS, PCOS-IR, Metformin, EVs, Microbial community, Full-length 16S rRNA

## Abstract

**Introduction:**

This study investigated changes in plasma microbial-derived extracellular vesicles (EVs) in patients with polycystic ovary syndrome and insulin resistance (PCOS-IR) before and after metformin treatment, and aimed to identify bacterial taxa within EVs that were biologically and statistically significant for diagnosis and treatment.

**Methods:**

The case–control study was conducted at Xiamen Chang Gung Hospital, Hua Qiao University. Plasma samples were collected from five PCOS-IR patients of childbearing age before and after 3 months of metformin treatment, and the samples were sequenced. The diversity and taxonomic composition of different microbial communities were analyzed through full-length 16 S glycosomal RNA gene sequencing.

**Results:**

After metformin treatment, fasting plasma glucose levels and IR degree of PCOS-IR patients were significantly improved. The 16 S analysis of plasma EVs from metformin-treated patients showed higher microbial diversity. There were significant differences in EVs derived from some environmental bacteria before and after metformin treatment. Notably, *Streptococcus salivarius* was more abundant in the metformin-treated group, suggesting it may be a potential probiotic.

**Discussion:**

The study demonstrated changes in the microbial composition of plasma EVs before and after metformin treatment. The findings may offer new insights into the pathogenesis of PCOS-IR and provide new avenues for research.

**Supplementary information:**

The online version contains supplementary material available at 10.1186/s13048-024-01444-x.

## Introduction

Polycystic ovary syndrome (PCOS) is characterized by chronic anovulation, hyperandrogenism and insulin resistance (IR). It is a complex endocrine and metabolic disorder, the pathogenesis of which is still unknown [1, 2]. In recent years, studies on humans and rodents have shown that microorganisms play a role in PCOS onset and development [3, 4]. For example, female genital tract dysbiosis and its possible impact on pathologies such as endometriosis, polycystic ovary syndrome (PCOS), pelvic inflammatory disease (PID), and gynecological cancers have been unveiled [5, 6]. Significant imbalances in gut flora have been observed in PCOS patients [7–10]. The structural and functional characteristics of the intestinal microbial community in patients with PCOS accompanied by IR (PCOS-IR) differ from those in PCOS patients without insulin resistance [11]. In a mouse model, mice transplanted with stools from PCOS patients developed IR [12]. Accordingly, it is speculated that the intestinal microbiota may be involved in the pathogenesis of PCOS by causing systemic inflammation and IR, altering the gut–brain axis and sex hormones [13].

Metformin alone can improve IR in PCOS patients and increase the ovulation rate in women with PCOS [14, 15]. The use of metformin in assisted reproductive technology improves live birth rates in women with gonadotropin-stimulated ovulation [16]. Microbial changes are common in PCOS patients during metformin treatment [17]. Evidence exists that changes in microbial metabolism induced by metformin contribute to improved therapeutic efficacy [18, 19]. Metformin treatment for PCOS can significantly increase the abundance of Lactobacilli flora and reduce IR, and changes in intestinal microorganisms are related to improvements in metabolism [20–22].

Extracellular vesicles (EVs) are substances secreted by prokaryotes and eukaryotes for intercellular communication [23]. EVs can carry a large number of diverse substances, including messenger RNA, microRNA (miRNA), proteins and lipids [24, 25]. In recent years, studies have found that specific miRNAs and proteins in serum EVs may play a role in the development of PCOS [26]. For example, miR-27a-5p and miR-424-5p in serum exosomes of PCOS patients may be involved in the process of abnormal follicle development [27, 28]. Programmed cell death protein 4 (PDCD4) is targeted by exosomal miR-323-3p to increase cumulus cell proliferation and inhibit apoptosis in PCOS [29]. Furthermore, cell transfer experiments with a functional circLDLR assay and its withdrawal in human granulosa cells have shown that depleting circLDLR in exosomes increases the expression of miR-1294 and inhibits the expression of cytochrome P450 family 1 subfamily A member 1 (CYP19A1) in recipient cells, reducing their estrogen secretion [30]. In addition, 86 differentially expressed proteins were found between patients with and without PCOS. The alterations in the proteomic profiles were related to inflammatory processes, reactive oxygen metabolism processes, cell migration and proliferation. S100 calcium-binding protein A9 (S100-A9) was found in follicular fluid exosomes. Nuclear factor-kappa B signaling is activated by exosome-enriched S100-A9 to enhance inflammation and disrupt steroidogenesis [31]. As extracellular messengers, serum EVs play a significant role in ovarian development [32].

Body fluids contain EVs derived from microbes that also play a key role in the interaction between microbes and hosts [33]. For example, EVs secreted by bacteria can cross the intestinal epithelial barrier and distribute to target organs. The brain–gut axis is a two-way communication system between intestinal bacteria and the brain. Changes in intestinal flora communicated via the brain–gut axis may have a pathogenetic role in PCOS [34]. On the one hand, abnormal hormone secretion in PCOS leads to the reduction in alpha diversity, resulting in an imbalance in the intestinal microbiota [33]. On the other hand, IR and hyperinsulinemia are also caused by intestinal flora and their metabolites, which stimulate gut–brain peptide secretion and modulate inflammatory pathways [35]. EVs are involved in metabolic processes and affect a variety of physiological phenomena, such as cancer and autoimmune diseases [36]. However, there are currently no relevant research reports on microbial-derived EVs in PCOS patients.

In this study, we evaluated both EVs and biological indicators in the plasma of PCOS-IR patients before and after metformin treatment. Unlike previous studies that primarily focused on the direct effects of local microbial communities near the ovaries, we chose to investigate the regulatory role of bacterial extracellular vesicles in the treatment of PCOS-IR. We conducted a comprehensive study of the diversity and abundance of different microbial communities in plasma EVs using full-length 16 S metagenomic sequencing. Our aim was to identify a bacterial classification group suitable for evaluating the effect of metformin treatment in PCOS-IR. This study can be used to find the role of gut microbiota in PCOS-IR and explore potential probiotic therapies.

## Materials and methods

### Sample collection

Plasma samples were collected from PCOS-IR patients of childbearing age before and after 3 months of metformin treatment. Patients received a dosage of 500 mg of metformin per day, administered in three divided doses every 8 h, for a continuous period of 12 weeks. A biochemical analyzer (i2000, Abbott, Chicago, IL, USA) was used to detect FPG (fasting plasma glucose) and HOMA-IR (homeostatic model assessment for insulin resistance).

The diagnostic criteria for PCOS refer to the 2018 edition of the Chinese guidelines for the diagnosis and management of polycystic ovary syndrome [37]. The criteria include oligomenorrhea or amenorrhea or irregular uterine bleeding, plus one of the following two items: (1) clinical manifestations of hyperandrogenism or hyperandrogenism; and (2) the appearance of polycystic ovarian morphology under ultrasound. We excluded patients with other diseases that may cause hyperandrogenism and other diseases that cause ovulation abnormalities. The diagnostic criteria for IR refer to an expert consensus on insulin resistance (2022 edition), and individuals with a HOMA-IR level ≥ 2.7 were considered to have IR [38, 39].

Recruitment conditions were as follows: (1) age > 18 years; (2) meeting PCOS diagnostic criteria; (3) agreement with biodatabase data collection and serological examination; and (4) HOMA-IR ≥ 2.7. The exclusion criteria were as follows: (1) patients who had not provided informed consent; (2) patients with a history of cardiovascular, stroke, and pulmonary diseases; (3) patients with mental illness; and (4) patients who had taken metformin, antibiotics, probiotics, antihistamines and steroids in the past 6 months.

### Ethics approval

The studies involving human participants were reviewed and approved by the Ethics Committee of Xiamen Chang Gung Hospital (approval number XMCGIRB2021024, approval date: September 13, 2021). Informed consent was obtained from the patients for participation in this study.

### Isolation of extracellular vesicles

EVs were isolated from blood samples by a series of ultrahigh-speed centrifugations. First, EDTA anticoagulated peripheral blood was centrifuged at 3000 × g for10min to obtain plasma. To remove debris from the plasma sample (4 mL), it was diluted five times with PBS and centrifuged at 2000×g for 30 min at 4 °C. A new tube was used for centrifuging at 10,000 × g for 45 min at 4 °C. The supernatant was filtered through a 0.45 μm syringe filter (R6BA09493, Millipore, Burlington, MA, USA), then ultracentrifuged at 100,000 × g for 70 min at 4 °C (CP100MX, Hitachi, Tokyo, Japan). Ultracentrifugation was performed again with resuspended EV pellets in 10 mL cold PBS. The final EV pellet was resuspended in 0.22 μm of filtered PBS for further analysis.

### Transmission electron microscopy

Transmission electron microscopy (TEM) was carried out with a negative stain to examine the morphology of isolated EVs. A 10 µL sample of purified exosomes was dropped on a copper grid for 1 min. Filter paper was used to remove excess EV solution. Staining the adsorbed EVs with ureanyl acetate for 1 min and removing excess liquid with filter paper was performed. A TEM image (HT-7700, Hitachi, Tokyo, Japan) at 100 kV was captured after the grids were dried at room temperature for several min.

### Nanoflow cytometry

Plasma EVs were analyzed for particle concentration and particle size using a Nano Analyzer (N30E, NanoFCM Inc., Xiamen, China) according to the manufacturer’s instructions. Appropriate amounts of each sample were diluted, and 30 µL of each diluted sample was added to 20 µL of anti-mouse CD9 and CD81 FITC Monoclonal antibodies (BD Biosciences, Franklin Lakes, NJ, USA). Isotype antibody IgG in flow cytometry experiments was used as a negative control (BD Biosciences, Franklin Lakes, NJ, USA). Two PBS washes were performed after incubation, and the mixture was centrifuged and kept at 4 °C for 70 min after incubation (CP100MX, Hitachi High-Technology Co., Ltd., Minato-ku, Tokyo, Japan). The supernatant was discarded, and the pellet was resuspended in 50 µL cold PBS and then tested on the machine.

### Western blot

The protein concentration of EVs was assessed using a BCA protein quantification kit (Beyotime, Haimen, China) following the manufacturer’s instructions. Bovine plasma albumin was used as a standard, and 5 µL of the sample was added to the BCA mixture and mixed. Incubation was carried out at 37 °C for 30 min, after which the absorbance value at OD562 nm was measured using a microplate reader (Varioskan LUX, Thermo, Waltham, MA, USA). The protein concentration of the sample to be tested was calculated based on the standard curve, and we used EV-labeled antibodies against goat CD9 (A1703, 1:1000, ABclonal, Wuhan, China), CD63 (ab134045, 1:1000, Abcam, Cambridge, UK) and calnexin (12,186, 1:1000, SAB, MD, USA). Anti-goat IgG peroxidase conjugate (AP132P, 1:5000, Merck Millipore, Burlington, MA, USA) was used for western blot testing. We loaded 10 µg of total protein per indicator into a 12% SDS‒PAGE gel based on the molecular weight of the target protein.

After gel running, the separation gel was transferred to a methanol-activated polyvinylidene fluoride membrane (Merck Millipore, Burlington, MA, USA) for “sandwich” transfer. After the transfer was completed, the polyvinylidene fluoride membrane was removed and soaked with the protein side up in 5% skim milk TBST and blocked for 1 h. Then, the membrane was incubated with primary antibodies overnight at 4 °C. Through three washes with TBST, excess primary antibodies were removed. Next, the membrane was incubated with the secondary antibody for 1 h at room temperature, and an equal volume of ECL A/B solution (1,810,212, Chemisignal ECL Plus, Shanghai, China) was dropped onto the polyvinylidene fluoride membrane and allowed to react in the dark for 5 min. Detection was performed using a chemiluminescence gel imaging system (3300 mini, ChemiScope, Clinx, Shanghai, China).

### Extraction of DNA from plasma EVs

DNA from plasma EVs was extracted using an EV DNA isolation kit (EXODNA50C-1, Runji, Liaoning, China). The extraction process referred to the instructions provided in the reagent kit manual. A 1 µL sample of the extracted DNA was used to measure the absorbance value on a multifunctional microplate reader (Varioskan LUX, Thermo, MA, USA) and calculate the concentration.

### Library creation and sequencing

We synthesized specific primers with barcodes according to the specified sequencing region. PCR was carried out using the following materials: TransGen AP221-02: TransStart® FastPfu DNA Polymerase, Beijing, China; PCR instrument: GeneAmp® 9700 type, ABI, Carlsbad, CA, USA. We performed three replicate experiments for each sample, mixing PCR products of different samples and incubating them with 2% agarose under formal experimental conditions. For gel electrophoresis detection, we used the AxyPrepDNA gel (AXYGEN Company, Carlsbad, CA, USA) recovery kit to cut the gel, recover the PCR products, and elute with Tris-HCl buffer. The QuantiFluor^TM−ST^ (Promega Company, Madison, WI, USA) blue fluorescence quantification system was used to detect and quantify the PCR products following electrophoresis. Sequencing libraries were generated using SMRTbell prep kit 3.0 (Pacific Biosciences, Menlo Park, CA, USA). Libraries were sequenced on the PacBio platform.

### Bioinformatics analysis process

PacBio off-machine data were obtained by using the ccs module of PacBio SMRTLink v11.0 analysis software to obtain hifi read (or ccs read) sequence files in fastq format. After distinguishing the samples by barcode, the correct sequence is identified based on the sequencing primer information, the sequence direction is corrected, and the primer sequence is removed. Through clustering operations, sequences are divided into many groups according to their similarities, and one group is an operational taxonomic unit (OTU). All sequences were divided into OTUs, and taxonomic analysis of OTU representative sequences with a 97% similarity level was performed using the RDP classifier Bayesian algorithm (software platform: Usearch, version 11 http://drive5.com/uparse/). Next, each sample’s community composition was counted at each classification level: domain, kingdom, phylum, class, order, family, genus, and species. The comparison databases used were as follows (the Silva database is used by default if not specified): Silva (Release138 http://www.arb-silva.de); RDP (Release 11.5 http://rdp.cme.msu.edu/); and Greengene (Release 13.5 http://greengenes.secondgenome.com/). We conducted an in-depth statistical and visual analysis of community structure and phylogeny based on the above analysis.

### Statistical analysis

The statistical analysis was conducted using version 8.0 of GraphPad Prism (GraphPad Software, San Diego, CA, USA). Data are expressed as the mean ± standard deviation. Differences between groups were assessed using the Wilcoxon rank-sum test. Statistical significance was considered when the two-sided p value was less than 0.05.

## Results

### Blood sugar levels and insulin resistance were improved after treatment

**Fig. 1:**
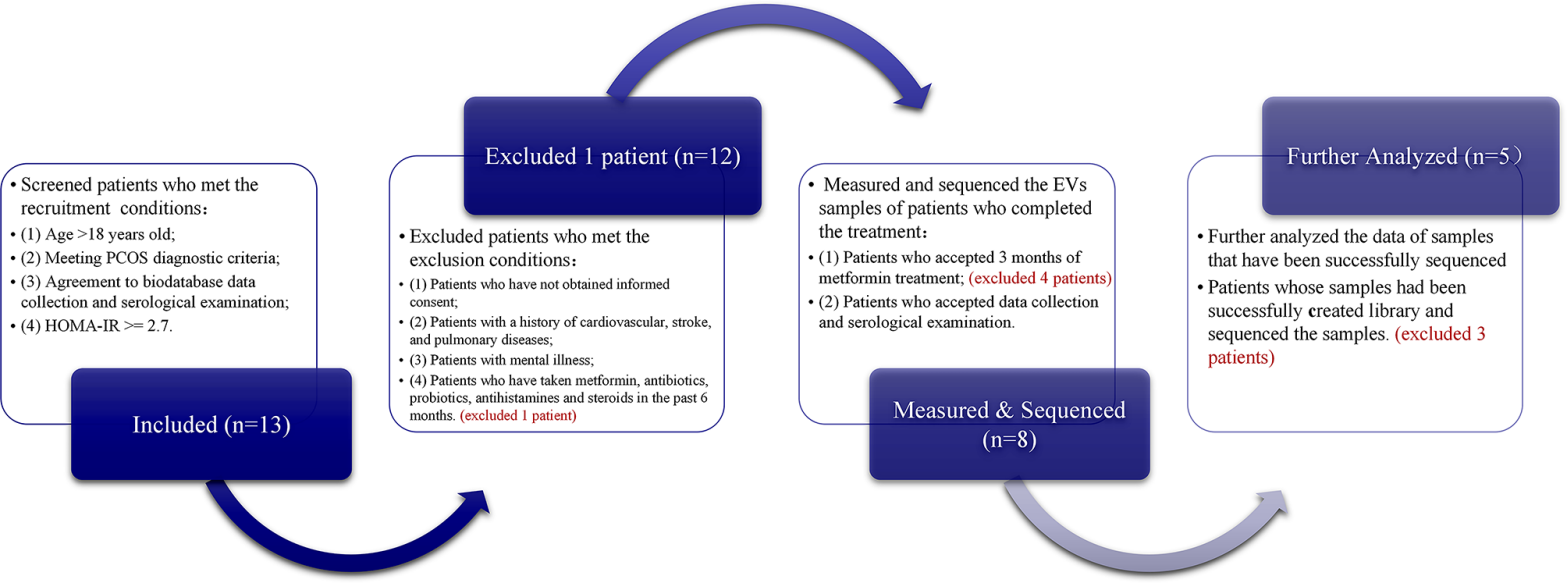
Recruitment and screening process of cases. A total of 13 patients met the inclusion criteria, but one patient was excluded because they had taken metformin within the past 6 months. Of the remaining 12 patients, four dropped out and did not complete the treatment. Among the eight samples obtained after 3 months of metformin treatment, three failed to be sequenced. Finally, five cases of PCOS-IR were collected. PCOS, polycystic ovary syndrome; IR, insulin resistance; HOMA-IR, homeostatic model assessment for insulin resistance; EVs, extracellular vesicles

We recruited 13 patients from March to May 2022 to participate in this study according to the recruitment criteria. Excluded one patient who has taken metformin, antibiotics, probiotics, antihistamines and steroids in the past 6 months.However, four patients dropped out and did not complete the treatment. Among the eight samples obtained after 3 months of metformin treatment, three failed to be sequenced. Thus, five cases of PCOS-IR were finally collected and divided into BF (before metformin treatment) and AF (after metformin treatment) **(**Fig. 1**)**. The characteristics of the case patients are shown in Table 1. All 5 patients had varying degrees of hyperglycemia and IR before treatment. After metformin treatment, FPG levels and the degree of HOMA-IR and Hyperandrogenemia of the five patients significantly improved (*p* < 0.05).

**Table 1:** Sample characteristics of PCOS-IR before and after treatment

	Age, year 32 (27, 37.5)	FBG, mmol/L		HOMA-IR		BMI		TEST		E2	
		BF	AF	BF	AF	BF	AF	BF	AF	BF	AF
		5.72 (5.64, 5.97)	5.38 (5.30, 5.59)	5.54 (4.80, 8.52)	3.93 (3.11, 4.27)	27.30 (26.90, 28.20)	27.10 (26.70, 28.00)	1.71 (1.53, 1.81)	1.54 (1.36, 1.59)	0.12 (0.12, 0.12)	0.24 (0.11, 0.49)
Patient-1	32	5.72	5.30	8.52	3.11	27.30	26.70	1.53	1.36	0.12	0.11
Patient-2	28	5.64	5.17	5.54	4.27	28.20	28.00	1.81	1.59	0.12	0.49
Patient-3	36	6.04	5.59	10.07	7.69	34.10	31.93	1.22	0.87	0.10	0.24
Patient-4	39	5.63	5.38	4.80	2.30	26.80	24.30	1.71	1.54	0.13	0.65
Patient-5	26	5.97	5.66	4.78	3.93	26.90	27.10	2.42	2.02	0.12	0.10
P Value		0.043		0.043		0.104		0.042		0.225	

Data are expressed as value or median (interquartile range)

FBG, fasting plasma glucose; HOMA-IR, Homeostatic Model Assessment for Insulin Resistancel; BMI, Body Mass Index; TEST, Testosterone; E2, Estradiol

BF, before metformin treatment; AF, after metformin treatment

### The separation and identification of plasma EVs

**Fig. 2:**
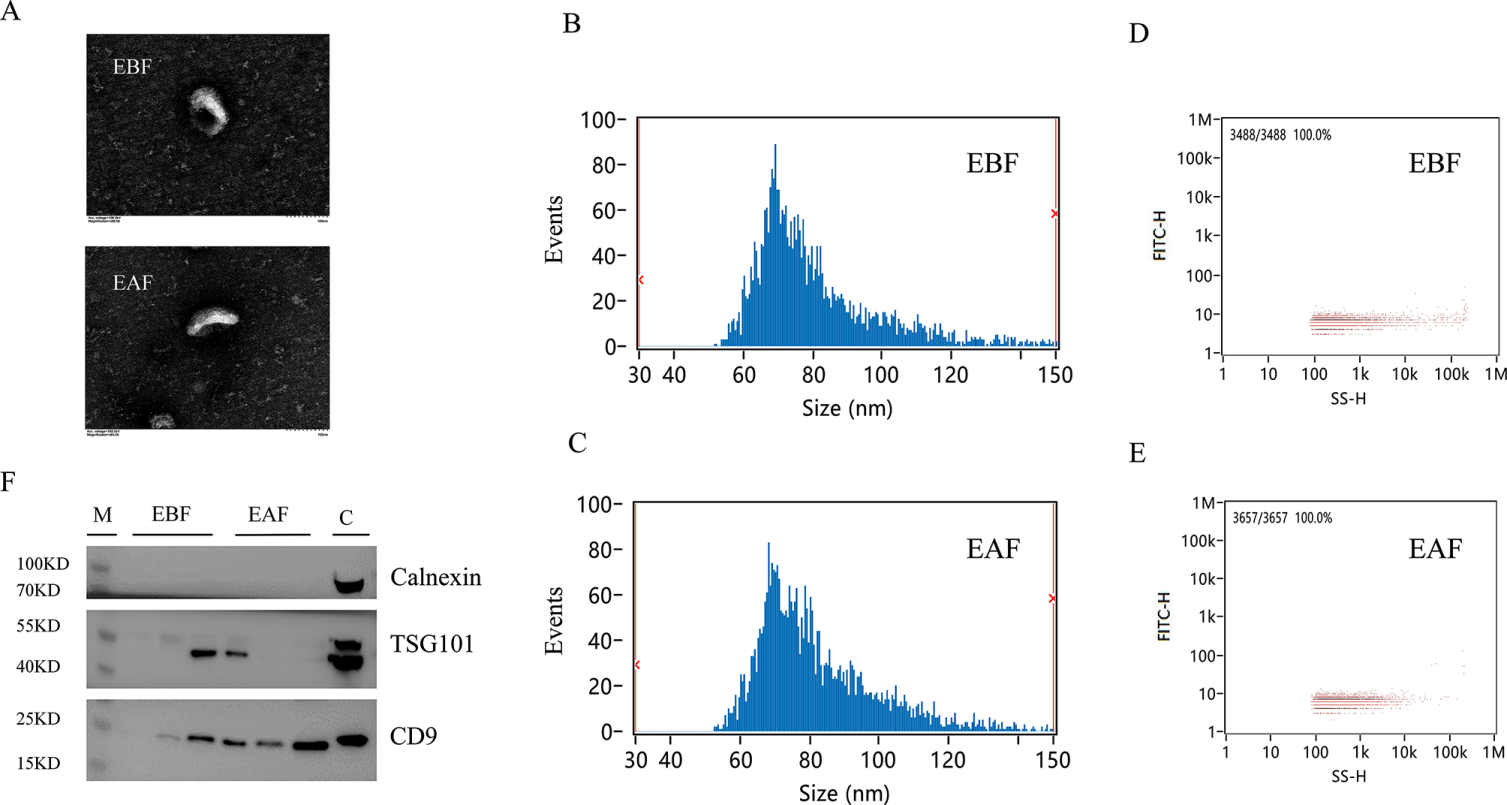
Isolation and identification of extracellular vesicles (EVs) from plasma. Electron microscopy images of EVs were observed through a transmission electron microscopy (stripes = 100 nm) **(A).** The particle size and concentration of EVs were measured using nanoflow cytometry **(B-E).** Western blot analysis was used to detect the expression of EV protein markers CD9, TSG101, and the expression of a non-EV marker calnexin, with 293T cells used as a negative control **(F)** (M: Marker; C: 293T cells; EBF: before metformin treatment; EAF: after metformin treatment)

Plasma EVs were isolated from PCOS-IR patients before and after metformin treatment, divided into an EBF group (EVs extracted from patients before treatment) and an EAF group (EVs extracted from patients after treatment), and identified and confirmed by TEM, nFCM and western blot analysis. Typical cup-shaped vesicles in EVs were identified by TEM (Fig. 2A). There was no significant difference in nanoparticle size or concentration between the two groups (Fig. 2B). The average sizes were 82.21 ± 2.40 nm for the EBF group and 83.36 ± 1.92 nm for the EAF group (Fig. 2B, C). After standardization, the particle concentrations were 1.75 ± 0.17 × 10^9^ particles/mL for the EBF group and 4.55 ± 0.91 × 10^9^ particles/mL for the EAF group (Fig. 2D–E). Western blot assays were used to assess the purity of the isolated EVs. Compared with the 293T cell control, protein markers of EVs, such as CD9 and TSG101, were detected, while the non-EV marker calnexin was not detected (Fig. 2F). In conclusion, the series of centrifugation processes were effective for the isolation of EVs.

### The EAF group exhibited higher microbial diversity

**Fig. 3:**
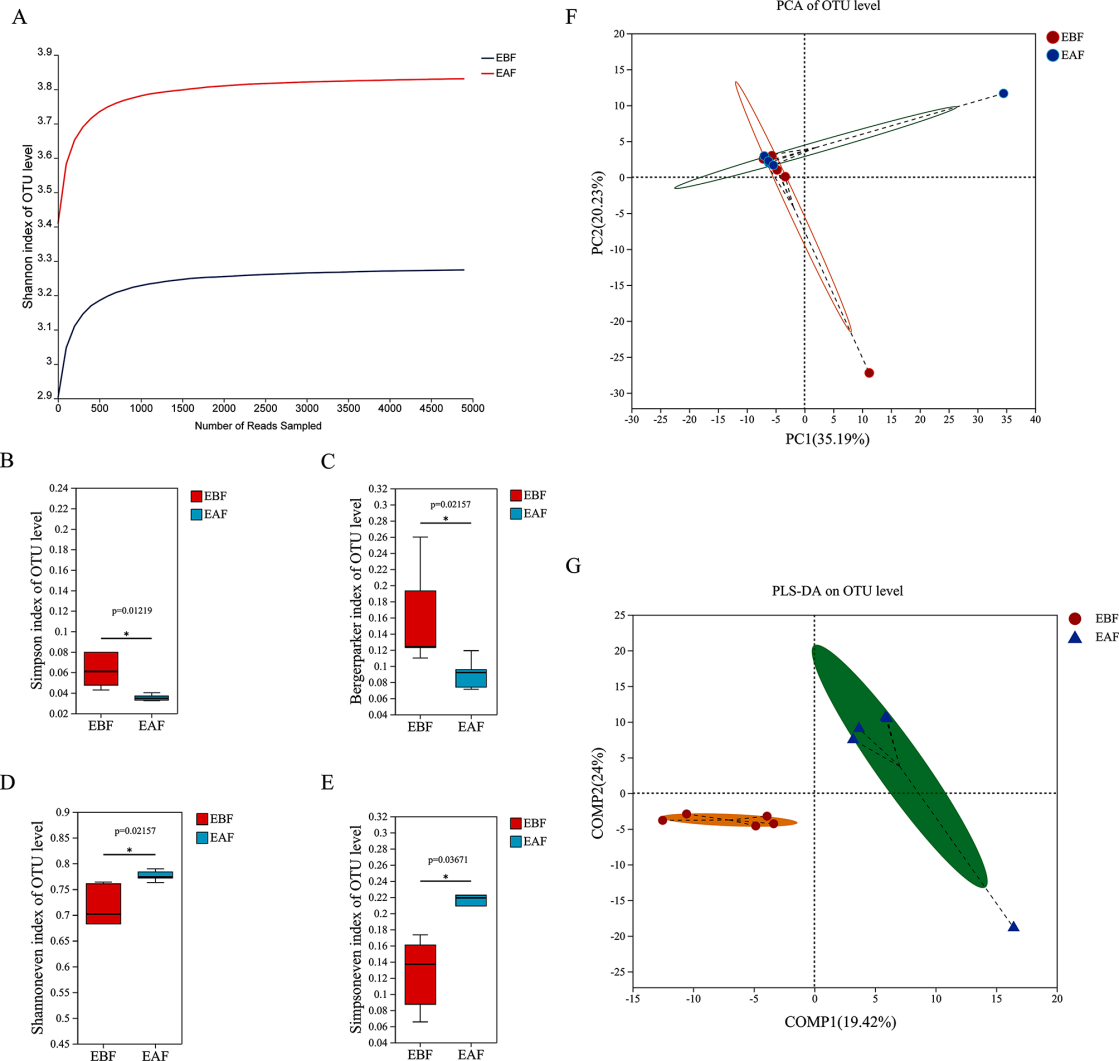
Analysis of bacterial community diversity of plasma extracellular vesicles in polycystic ovary syndrome patients before and after metformin treatment. The sequencing volume was evaluated through the Shannon index dilution curve **(A)**, and the alpha diversity of the two groups was evaluated by box plots of the Simpson index **(B)**, BergerParker index **(C)**, Shannoneven index **(D)** and Simpsoneven index **(E).** The beta diversity of plasma microbial communities in each group was analyzed by principal component analysis (PCA) **(F)** and partial least squares discriminant analysis (PLS-DA) **(G).** (EBF: before metformin treatment; EAF: after metformin treatment; OTU: operational taxonomic unit)

We sequenced the different microbial communities between the EBF group (*n* = 5) and the EAF group (*n* = 5). The unqualified sequences were removed, a total of 431,454 valid sequences were generated, with each sample generating an average of 43,145 ± 16,290 valid sequences (valid sequence range: 13,521 to 60,170). Shannon index dilution curve analysis showed that the observed bacterial species reached a plateau (Fig. 3A). The alpha diversity index showed that the Simpson index and Berger-Parker index were significantly different between the EBF group and the EAF group (*p* < 0.05), and the EAF group showed higher richness (Fig. 3B–C). The Shannoneven and Simpsoneven indexes were significantly different between the EBF group and the EAF group (*p* < 0.05), such that the EAF group showed higher evenness (Fig. 3D–E). Beta diversity analysis was performed to estimate the similarity of microbial communities between groups. Principal component analysis showed that the EBF and EAF groups accounted for 35.19% and 20.23% of the total variation, respectively **(**Fig. 3F). The partial least squares discriminant analysis plot showed that there were differences between the two groups, with total variations of 19.42% and 24% in PLS1 and PLS2, respectively (Fig. 3G). These results collectively indicate that there are differences in microbial communities between the EBF group and the EAF group, with the EAF group exhibiting higher microbial diversity.

### Distribution of dominant bacteria at different taxonomic levels

**Fig. 4:**
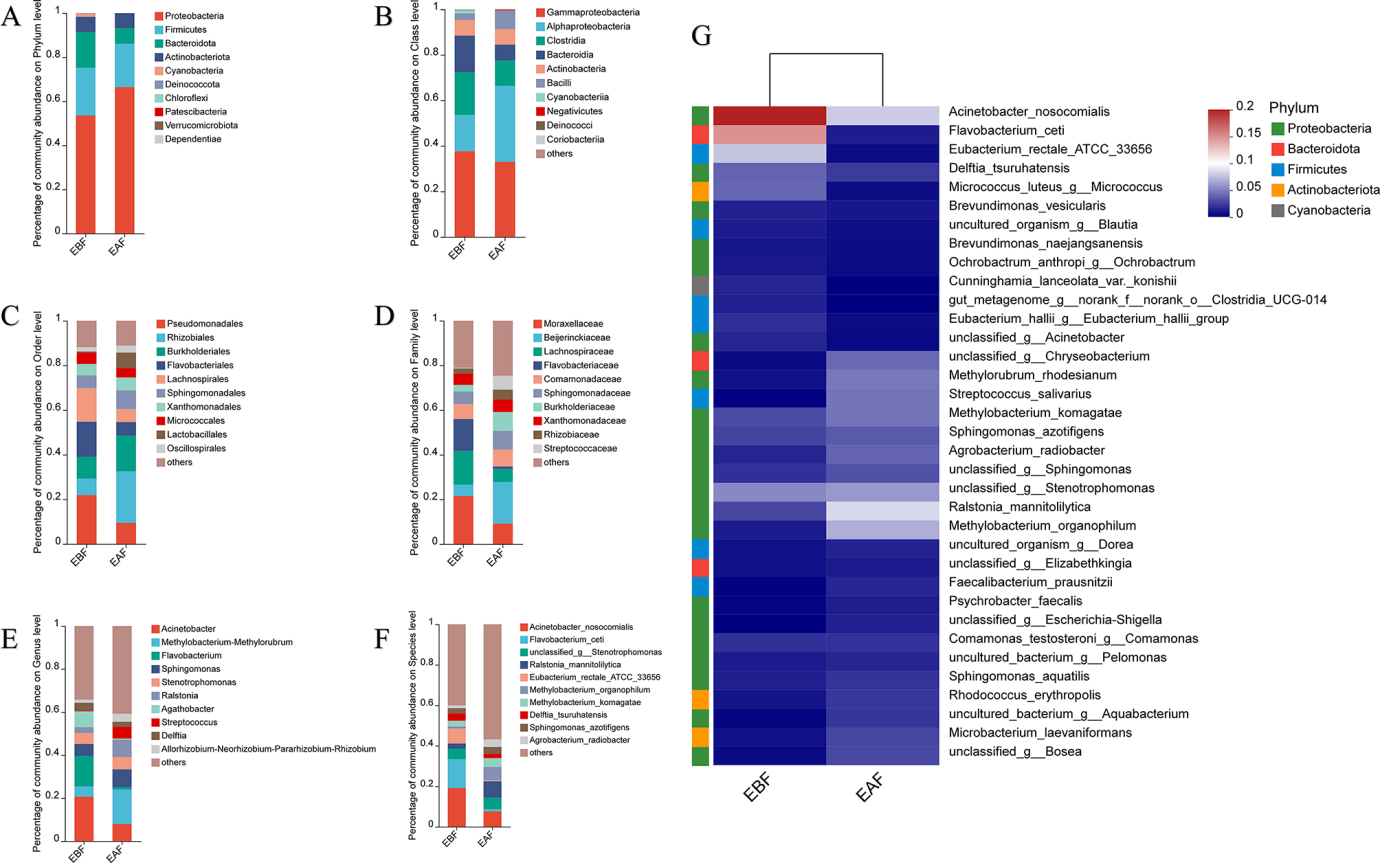
The distribution of the relative abundance of the top 10 species in the two groups (EBF: before metformin treatment; EAF: after metformin treatment) at the phylum **(A)**, class **(B)**, order **(C)**, family **(D)**, genus **(E)**, and species **(F)** levels. The horizontal axis represents the sample name and the vertical axis represents the proportion of the species in that sample. Different colors of the bars represent different species, and the length of the column represents the percentage of the species. Heat map of 35 bacterial species **(G)**

**Fig. 5:**
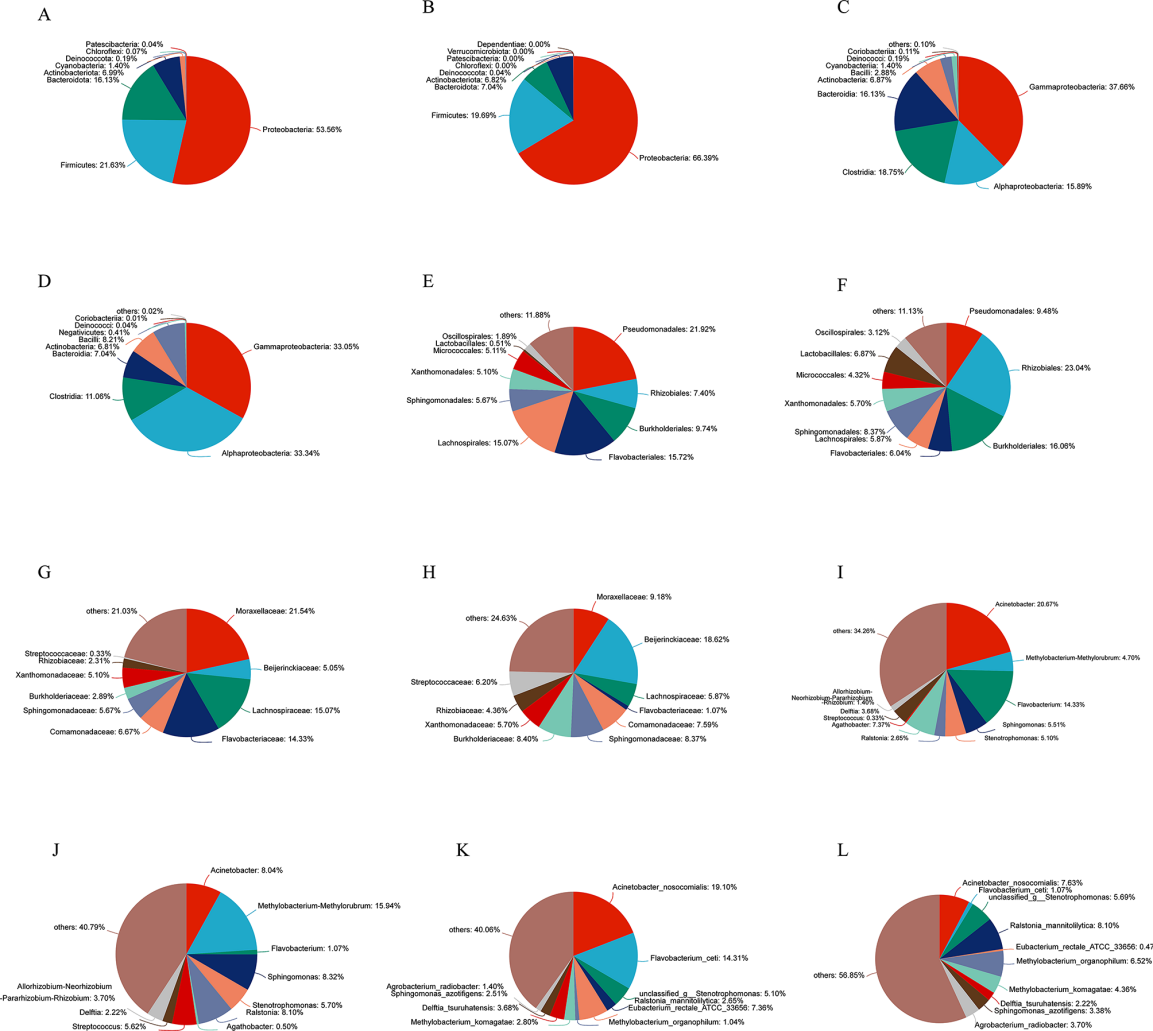
Pie charts of the relative abundance of the top 10 species in the two groups (EBF: before metformin treatment; EAF: after metformin treatment) at the phylum (**A**: EBF; **B**: EAF), class (**C**: EBF, **D**: EAF), order (**E**: EBF, **F**: EAF), family (**G**: EBF, **H**: EAF), genus (**I**: EBF, **J**: EAF) and species (**K**: EBF, **L**: EAF) levels

A total of 502 OTUs were obtained, which were taxonomically associated with 10 phyla, 14 classes, 45 orders, 84 families, 157 genera, and 265 species (TableS1). By evaluating the ratio of the top 10 bacterial species at each taxonomic level, the dominant bacteria were determined (Figs. 4 and 5). At the phylum level, the dominant bacteria were *Proteobacteria* and *Firmicutes*, with *Bacteroidota* and *Cyanobacteria* having higher abundance percentages in the EBF group and *Proteobacteria* having higher abundance percentages in the EAF group (Fig. 4A). At the class level, the dominant bacteria were *Gammaproteobacteria* and *Alphaproteobacteria*, with *Gammaproteobacteria* and *Bacteroidia* having higher abundance percentages in the EBF group and *Alphaproteobacteria* and *Bacilli* having higher abundance percentages in the EAF group. (Fig. 4B). At the order level, *Pseudomonadales* and *Xanthomonadales* were more abundant in the EBF group, while *Rhizobiales*, *Burkholderiales* and *Lactobacillales* had a higher abundance percentage in the EAF group (Fig. 4C). At the family level, *Moraxellaceae*, *Lachnospiraceae* and *Flavobacteriaceae* had higher percentages in the EBF group, while *Xanthomonadaceae*, *Bayerinkellaceae*, *Streptococcusaceae*, *Burkholderiaceae*, *Sphingomonadaceae* and *Rhizobiumaceae* had higher abundance percentages in the EAF group (Fig. 4D). At the genus level, *Acinetobacter*, *Flavobacterium* and *Agarobacterium* were more abundant in the EBF group, while *Methylobacterium*, *Sphingomonas*, *Ralstonia*, *Streptococcus* and *Rhizobium* were more abundant in the EAF group (Fig. 4E). At the species level, *Acinetobacter nosocomialis*, *Xanthomobacterium cetaceans* and *Eubacterium rectal* were more abundant in the EBF group, and *Ralstonia mannitoligenes*, *Methylobacterium organophilum* and *Streptococcus salivarius* were more abundant in the EAF group (Fig. 4F). The heatmap of the top 35 bacterial species showed differences between the microbial communities of the two groups (Fig. 4G). The percentages of dominant bacteria at different levels are shown in Fig. 4 and Appendix Table S2.

### Analysis of differences in microbial community composition

**Fig. 6:**
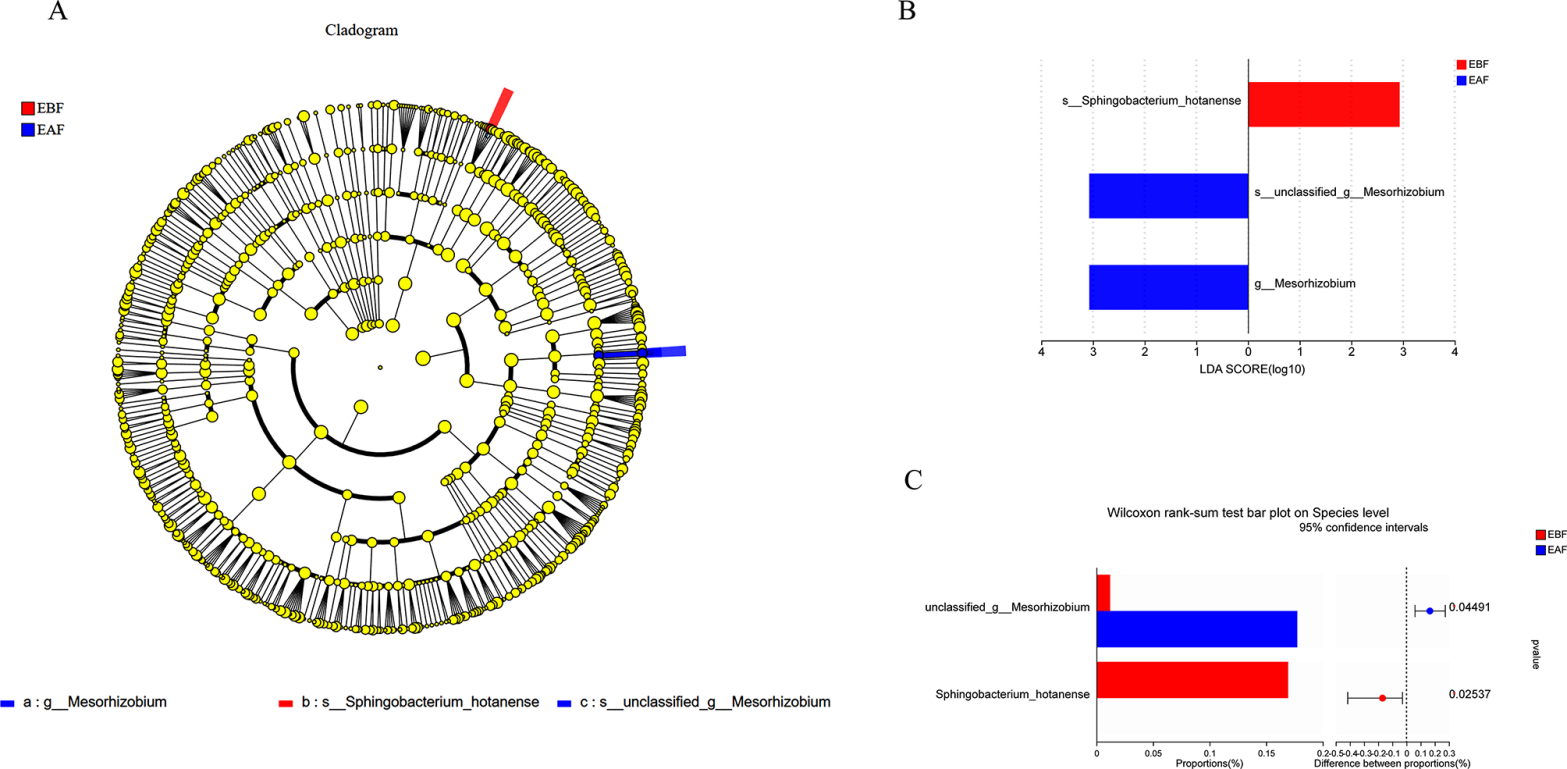
Analysis of microbial species diversity. Microbial cladogram **(A)**: In the cladogram, the circles radiating from the inside out represent the taxonomic levels from the phylum level to the genus (or species) level. Each small circle at a different taxonomic level represents a taxon of that category, the diameter of the small circle is yellow for species with no significant difference in relative abundance, blue nodes represent microbial communities that play an important role in the EAF group, and red nodes represent the microbial communities that play an important role in the EBF group. Histogram of LDA value distribution **(B)**: the length of the histogram represents the impact size (LDA score) of different bacterial species. Specieslevel difference test plot between groups (C, Wilcoxon rank-sum test): the length of the histogram represents the proportion of different bacterial species (EBF: before metformin treatment; EBF: after metformin treatment)

LEfSe with a log LDA score cut-off of > 2.0 was used to compare the predicted microbial taxa of the EBF and EAF groups. The cladogram showed that the number of significantly different communities between the two groups was close (Fig. 6A). Histograms of LDA scores and intergroup difference analysis (*p* < 0.05) showed that the abundance percentage of EVs derived from *Sphingobacterium hotanense* was higher in the EBF group; the abundance percent of EVs derived from *Bradyrhizobium* was higher in the EAF group (Fig. 6B–C).

## Discussion

Our results show that after treatment with metformin, the FPG levels and IR degree of PCOS-IR patients were significantly improved. The 16 S analysis of plasma EVs from metformin-treated patients showed higher microbial diversity, and the distribution of dominant bacteria at each level was different from that before treatment **(**Figs. 4 and 5**)**. Among these differences, there was a large difference in the distribution of *Streptococcus salivarius* in the *Lactobacillus* order before and after treatment. In addition, there were significant differences in EVs derived from some environmental bacteria before and after metformin treatment.

Studies have confirmed that EVs play a key role in intercellular communication between the host and commensal microorganisms [40]. More active bacteria can secrete more EVs, and observing changes in the exosomes of microorganisms can more accurately reflect the role of microorganisms in the host than simply looking at changes in microbial volume. Heo et al. [41] found that analysis of gut-microbe-derived EVs was better at differentiating *patients with Inflammatory Bowel Disease* (IBD ) patients from healthy controls than fecal microbiome analysis. PCOS is an endocrine disease involving systemic metabolic abnormalities. The intestinal microbiota is related to the occurrence and development of PCOS-IR. Therefore, detecting blood EVs can help reflect the role of microorganisms in the development of PCOS-IR. Studies have found that the diversity of the intestinal flora in PCOS-IR patients is reduced [13], and this study observed an increase in the microbial diversity of EVs in PCOS-IR patients after metformin treatment. Previous studies have shown that EVs derived from Proteobacteria are more abundant in the blood, while EVs derived from Firmicutes and Bacteroidetes are more abundant in the intestines [42]. This study found the following order in terms of the percent of EVs at the phylum level: Proteobacteria > Firmicutes > Bacteroidetes > Actinobacteria **(**Fig. 4, **Table ****S2****)**. Based on this, we speculate that the EVs isolated from plasma in our study may partly have come from intestinal bacteria. Among them, *Bacteroidetes* had a higher abundance ratio in the EBF group, while *Firmicutes butyrate-producing Agatobacterium* and *Eubacterium* rectal-derived EVs had a higher abundance ratio in the EBF group, which is consistent with previous reports of different gut microbiome profiles [43, 44]. In their study, Heo et al. [41] found that the microbiota that caused significant differences between the IBD group and the control group differed in the fecal microbiome and gut microbe-derived EVs.

In this study, the percent abundance of EVs derived from *Pseudomonas* in the EBF group was higher than that in the EAF group. The main relevant bacterial species was *Acinetobacter nosocomialis*, which is considered to cause colonization due to the medical treatment process. The main related bacterial species in the EBF group with a high abundance percent of *Flavobacteriales* was *Cetobacterium somerae*, which is a marine bacterium [45]. Considering that the hospital is located in a coastal city, it was presumed to be contact colonization. The percent abundance of EVs derived from *Rhizobiales, Burkholderiales* and *Lactobacillales* in the EAF group was higher. In particular, the percentage of *Lactobacillus*-derived EVs was very low in the EBF group (0.51%), and its percentage increased to 6.87% after metformin treatment. Further analysis revealed that the main related species with a high percent abundance of *Lactobacilliles* was *Streptococcus salivarius***(**Fig. 4, **Table ****S2****)**. After metformin treatment, the abundance percent of EVs derived from *Streptococcus salivarius* in the blood of PCOS-IR patients increased from 0.03 to 4.33%.

*Streptococcus salivarius* is a member of the genus Streptococcus, is often detected in fermented dairy products and is commonly used in the production of yogurt. Dolatkhah et al. [46] conducted a double-blind, placebo-controlled randomized clinical trial on 64 patients with gestational diabetes mellitus. After supplementation with a probiotic oral solution containing *Streptococcus thermophilus* (*Streptococcus salivarius subsp*. *Thermophilus*) (*Lactobacillus acidophilus* LA-5, *Bifidobacterium BB-12, Streptococcus thermophilus* STY-31, and *Lactobacillus bulgaricus LBY-2 biocapsules* > 4 × 10^9^ CFU), the weight gain in the probiotic group was significantly lower than that in the placebo group (*p* < 0.05). FPG decreased significantly in both the intervention and control groups, with a greater decrease in the probiotic group (*p* < 0.05). Moreover, the HOMA-IR of the probiotic group decreased by 6.74% during the study period (*p* < 0.05). Further analysis by the research team found significantly higher levels of serum high-sensitivity C-reactive protein and tumor necrosis factor-α in the probiotic group than in the placebo group. In addition, malondialdehyde, glutathione reductase and red blood cell glutathione peroxidase levels were significantly increased after using probiotics, suggesting that these probiotic supplements improve several biomarkers of inflammation and oxidative stress in women with gestational diabetes mellitus [47]. Ma et al. studied the relationship between body mass index and fecal *Streptococcus* infection in adults in Xining City. They found that the abundance of intestinal *Streptococcus salivarius* was negatively correlated with human body mass index, being a major determinant of metabolic regulation and mild inflammation of adipose tissue in the gut microbiota and a potential probiotic [48]. These studies collectively demonstrate the impact of *Streptococcus salivarius* on human glucose and lipid metabolism. PCOS includes genetic and epigenetic susceptibility, hypothalamic and ovarian dysfunction, excessive exposure to androgens, insulin resistance, and obesity-related mechanisms [49]. Based on the results of this study, we speculate that increasing the activity of *Streptococcus salivarius* may be one of the mechanisms by which metformin improves systemic metabolic symptoms in patients with PCOS-IR.

In this study, EVs derived from some environmental bacteria were found in blood EVs. Among them, *Xanthomonadaceae* is a plant pathogen [50], and *Beijerinellaceae* is a soil bacterium [51]. The percentage of EVs derived from these two bacteria in the EAF group was higher because of lifestyle colonization. The results of LEfSe analysis and intergroup difference analysis showed that there were significant differences in soil and plant-derived microorganisms such as *Sphingobacterium hotanense* and *Bradyrhizobium mesogenum*. The percentage of EVs derived from *Sphingobacterium hotanense* was higher in the EBF group (*p* < 0.05). *Sphingobacterium hotanense* is a new type of gram-negative bacterial strain isolated from the soil of the Populus euphratica forest in the Hotan Valley of the Xinjiang Uygur Autonomous Region of China [52]. To date, only one case of human infection has been reported [53]. The percentage of EVs derived from *Bradyrhizobium* in the EAF group was higher (*p* < 0.05). *Bradyrhizobium* is a growth-promoting bacterium for leguminous plants that functions in biological nitrogen fixation, and it cannot be ruled out that patients are colonized by dietary intake [54]. Whether it has similar or unknown effects in the human body remains to be further explored. The percentage of the above bacterial strains in the group was very low (< 0.2%), and their sources and functions need to be further explored by expanding the sample size and carrying out related animal model experiments in the future.

In the present study, we investigated the changes in bacterial 16 s in plasma EVs in patients with PCOS and insulin resistance (PCOS-IR) before and after metformin treatment, which may reveal a new perspective for elucidating the pathogenesis of PCOS-IR and provide new avenues for peer research.

Despite the small sample size affecting the statistical results, the study demonstrated changes in the microbial composition of blood plasma EVs before and after metformin treatment. The bacterial species with a large change in abundance percentages could serve as plasma biomarkers for therapeutic effects in PCOS-IR patients or become potential therapeutic probiotics. Based on these changes, further research can be conducted on the possible mechanisms of systemic regulation of PCOS-IR patients derived from characteristic microbial communities. In vivo experiments could involve the use of animal models, such as mice, to study the systemic effects of these microbial communities on the host organism, particularly focusing on metabolic pathways related to PCOS-IR. These experiments would provide a more comprehensive understanding of the role these microbial communities play in PCOS-IR.

As the first project to implement the measurement of plasma microbial-derived EVs in patients with PCOS-IR, our study also had some limitations related to clinical reality. For example, some EVs produced by intestinal microbes circulate throughout the body through the host’s colonic mucosa and vascular system and are eventually excreted through urine [55]. In future studies, measurements of EVs in an increased number of different source samples can be undertaken. These inherent limitations were unlikely to alter our main findings.

## Conclusions

In summary, this study explored the compositional characteristics of EVs derived from the blood microbiota in patients with PCOS-IR before and after metformin treatment. The results may help to clarify the role of the microbiome in PCOS-IR and may aid in diagnosis and treatment.

### Electronic supplementary material

Below is the link to the electronic supplementary material.

Supplementary Material 1

Supplementary Material 2

## Data Availability

Data relating to the metagenomic sequencing that support the findings of this study have been uploaded to the Sequence Read Archive database (https://trace.ncbi.nlm.nih.gov/Traces/home/) and are available for download via accession number PRJNA1073749.
