# Correction: Metformin modifies plasma microbial-derived extracellular vesicles in polycystic ovary syndrome with insulin resistance

**DOI:** 10.1186/s13048-024-01481-6

**Published:** 2024-07-27

**Authors:** Liping Hu, Guolin Hong, Jingzhi Li, Mengkun Chen, Chih-Jung Chang, Po-Jen Cheng, Zhimei Zhang, Xinli Zhang, Huiping Chen, Yingting Zhuang, Yuqin Li

**Affiliations:** 1 Department of Laboratory Medicine, Xiamen Chang Gung Hospital Hua Qiao University, Xiamen, 361028 P. R. China; 2 The Third Clinical Medical College, Fujian Medical University, Fuzhou, P. R. China; 3 Department of Laboratory Medicine, Xiamen Key Laboratory of Genetic Testing, The First Affiliated Hospital of Xiamen University, School of Medicine, Xiamen University, Xiamen, 361005 P. R. China; 4 Department of Obstetrics, Xiangya Hospital Central South University, Changsha, P. R. China; 5 Department of Gynecology and Obstetrics, Xiamen Chang Gung Hospital Hua Qiao University, Xiamen, P. R. China; 6 School of Medicine, Hua Qiao University, Quanzhou, P. R. China; 7 School of Pharmacy, Fujian Medical University, Fuzhou, P. R. China; 8 Medical Research Center, Xiamen Chang Gung Hospital Hua Qiao University, Xiamen, P. R. China; 9 Drug Hypersensitivity Clinical and Research Center, Department of Dermatology, Chang Gung Memorial Hospital, Taoyuan, Linkou, Taiwan

**Correction: J Ovarian Res 17, 136 (2024)**

10.1186/s13048-024-01444-x

Following publication of the original article [1], the authors reported that affiliations of author ‘Guolin Hong’ and affiliation 3 were incorrectly presented. The author group is presented correctly in this correction article.

The original article [1] has been corrected.
